# Short term storage stability at room temperature of two different platelet-rich plasma preparations from equine donors and potential impact on growth factor concentrations

**DOI:** 10.1186/s12917-016-0920-4

**Published:** 2017-01-05

**Authors:** Gregor Hauschild, Florian Geburek, Georg Gosheger, Maria Eveslage, Daniela Serrano, Arne Streitbürger, Sara Johannlükens, Dirk Menzel, Reinhard Mischke

**Affiliations:** 1 Department of Orthopedics and Tumororthopedics, University Hospital of Muenster, Albert-Schweitzer-Campus 1, 48149 Muenster, Germany; 2 Clinic for Horses, University of Veterinary Medicine Hannover, Foundation, Bünteweg 9, 30559 Hanover, Germany; 3 Small Animal Clinic, University of Veterinary Medicine Hannover, Foundation, Bünteweg 9, 30559 Hannover, Germany; 4 Institute of Biostatistics and Clinical Research, University of Muenster (WWU), Schmeddingstraße 56, 48149 Münster, Germany

**Keywords:** Regenerative medicine, Platelet-rich plasma, Platelet-derived growth factor-BB, Transforming growth factor ß1, Storage time, Applicability

## Abstract

**Background:**

The increasing interest in platelet-rich plasma (PRP) based therapies is as yet accompanied by inconsistent information regarding nearly all aspects of handling and application. Among these storage stability of processed platelet-rich products may be the basis for a more flexible application mode. The objective of this study was (1) to estimate the storage stability of growth factors platelet derived growth factor BB (PDGF-BB) and transforming growth factor ß1 (TGF-ß1) in both, a single-step softspin centrifugation-based pure-PRP (P-PRP, ACP®), and a gravity filtration system-based leukocyte-rich-PRP (L-PRP, E-PET), over a six hours time span after preparation at room temperature and (2) to identify possible factors influencing these growth factor concentrations in an equine model.

**Results:**

Growth factor concentrations remained stable over the entire investigation period in L-PRP as well as P-PRP preparations revealing a mean of 3569 pg/ml PDGF-BB for E-PET and means of 1276 pg/ml PDGF-BB and 5086 pg/ml TGF-ß1 for ACP®. Pearson correlations yielded no significant impact of whole blood platelet (PLT), white blood cell (WBC) and red blood cell (RBC) counts on resulting cytokine values. In case of ACP® no significant dependencies between PLT, WBC and RBC counts of the processed platelet-rich product and resulting cytokine content occurred with exception of TGF-ß1 concentrations showing a strong correlation with the WBC content. PDGF-BB content of E-PET preparations showed a strong positive correlation with PLT and a strong negative with WBC of these preparations but not with RBC.

**Conclusions:**

L-PRP ad modum E-PET and P-PRP ad modum ACP® are applicable over at least a six hours time span at room temperature without loss of growth factor content. Based on the results of this study factors influencing the resulting growth factor concentrations still remain questionable. Additional studies implicating a further standardization of preparation protocols are necessary to identify consistent impact on cytokine content after PRP processing.

**Electronic supplementary material:**

The online version of this article (doi:10.1186/s12917-016-0920-4) contains supplementary material, which is available to authorized users.

## Background

Platelet-rich plasma (PRP) – currently defined as a volume of the plasma fraction of autologous blood with concentrations of platelets above baseline values [1–3] – is being widely used as a factor-based approach in regenerative medicine. Since the late 1980s in fields including but not limited to cardiac surgery, wound care and plastic surgery, maxillofacial surgery and dentistry application of PRP-preparations has been evaluated and documented in numerous case-reports, clinical trials and experimental studies. In addition, over the last decade PRP-based therapies of musculoskeletal diseases, such as tendon and ligament lesions, muscle tears and muscle strain lesions [4], bone and cartilage lesions [5] as well as osteoarthritis [6], have been focus of special interest. Despite the large amount of data generated by experimental and clinical implementation of this approach in human and, to a lesser degree, in veterinary medicine, there is still a severe lack of evidence and consistency in all aspects, including preparation protocols, nomenclature, dynamics and kinetics of released growth factors and other cytokines, application protocols and others. With regard to human medicine literature challenges to be addressed include among others the complete characterization of the individual products and their released structures, determination of intra-individual impact on platelet-rich preparations’ efficiency and the development of novel interactions with biomaterials to expand the range of application [7]. Engebretsen and colleagues emphasized in an IOC (International Olympic Committee) consensus paper [8] the paucity of high-level clinical trials on application of PRP in human sports medicine in addition to very limited basic science data on the influence of PRP on the inflammation and repair of connective tissue and skeletal muscle. This is underlined by a lack of current industry consensus concerning dosage protocols [8, 9]. The fact that each of a multitude of different preparation techniques and protocols leads to a product with different biological properties and potential application fields [10, 11] makes it very difficult for both clinicians and scientists to choose the appropriate handling during preparation and application in their specific entity [12]. Thus, usage of three different devices for processing buffy coat PRP after obtaining whole blood from human donors revealed significantly different platelet content ranging from 2.6 to 5.6 fold concentration compared to baseline values and significantly different growth factor levels [13]. With regard to veterinary literature platelet concentrations obtained from equine donors and following a buffy coat method protocol were 8.9 fold higher than baseline concentrations [14]. Compared to whole blood platelet concentrates obtained from equine donors and processed by a double centrifugation tube method contained 1,21 to 1,7 fold higher platelet concentrations [15]. However PRP obtained from canine donors and processed by a double centrifugation tube method revealed platelet concentrations up to 4.9 fold higher than baseline values [16]. As far as growth factor content of the completed PRP-preparation is considered, critical for the intended enhancement of wound healing and regenerative capacities at site of the lesion, final concentrations at time of application are of particular interest. Early preparation protocols - such as PRGF® (Endoret® BTI, San Antonio, Spain) – have been intended for immediate use or usage within 30 min, respectively [10, 17], while recent studies on the biological stability of growth factor concentrations of preparations following this same basic protocol reveal a post-preparation stability at room temperature for 72 h [18]. For the single spin protocol of ACP® (Autologous Conditioned Plasma, Arthrex Vet Systems, Frechen, Germany) for example the manufacturers’ reference advises usage within 30 min after preparation if waiving the addition of an anticoagulant is intended and within 4 h if it is not [19] while in case of E-PET the manufacturer recommends usage within 60 min.

Based on these inconsistencies regarding procedures and handling, the objective of this study was (1) to estimate the storage stability of growth factors PDGF-BB (platelet-derived growth factor-BB) and TGF-β1 (transforming growth factor-ß1) in both, a single-step softspin centrifugation-based pure-PRP (ACP®), and a gravity filtration system-based leukocyte-rich-PRP (E-PET, Equine Platelet Enhancement Therapy, Pall GmbH, Dreieich, Germany) over a six hours time span after preparation at room temperature and (2) to identify possible factors that may influence the resulting growth factor concentrations in an equine model.

## Methods

Animal experiments were conducted under an ethics committee (Lower Saxony State Office for Consumer Protection and Food Safety) approved protocol in accordance with German federal animal welfare legislation (Az 33.14-42502-04-14/1547), which is in compliance with the guidelines outlined in the NRC Guide for the Care and Use of Laboratory Animals. All animals were from the ownership of and housed at the Clinic for Horses of the University of Veterinary Medicine, Hannover, Foundation, Germany. All procedures associated with the experimental animals were performed in this same facility.

### Test animals

Fifteen warm-blood horses (six mares, four stallions and five geldings) free from clinical signs of disease with a mean age of 8, 6 years (range: 2–22 years) were included in this study. Initial CBC data of these were within equine reference ranges given by the central laboratory.

### Study design

The study followed a prospective non-randomized protocol including two different PRP-processing systems (ACP®, E-PET) and one control group (physiological sodium chloride solution, NaCl). All measurements were carried out in a blinded fashion. As part of a superordinated experimental animal study, whole blood samples were taken from 15 adult horses (1) standing, non-sedated and (2) the following day under general anaesthesia to be intended for complete blood count (CBC).

Under this latter condition whole blood samples of these same 15 horses were obtained to prepare 15 doses of autologeous conditioned plasma ad modum ACP®. In addition whole blood samples from ten of these same 15 test animals were obtained to produce 10 doses of PRP ad modum E-PET. Five samples of sterile sodium chloride (NaCl) solution (B. Braun Melsungen AG, Germany) served as control. Being part of a superordinated experimental animal study the number of ACP® and E-PET preparations processed – and thereby the resulting ratio of 15 to 10 PRP doses - was due to the requirements of the overall design.

Following preparation of PRP preparations from each donor, and after control doses of NaCl had been obtained, an 0.5 ml aliquot of each of these was transferred into 2 ml Eppendorf tubes (Eppendorf Safe-Lock tubes, Eppendorf AG, Hamburg, Germany) and immediately intended for CBC. Further 0.5 ml aliquots were transferred into 2 ml cryopreservation tubes (CryoPure Röhren, Sarstedt AG & Co, Nümbrecht, Germany), containing 10 I.E. Heparin (ratiopharm GmbH, Ulm, Germany) and stored at room temperature.

Immediately (T_0_) after preparation or obtaining, respectively and after a storage period at room temperature of 1 (T_1_), 5 (T_5_), 30 (T_30_), 60 (T_60_), 180 (T_180_) and 360 (T_360_) minutes these samples were snap-frozen in liquid-nitrogen (Air Liquide Deutschland GmbH, Kornwestheim, Germany) at −196° Celsius (°C) to fix the respective sample status at the different points of time.

After storage at below – 80° Celsius the samples of both PRP protocols and control were thawed in a single modus in a water bath at 37 °C followed by measurement of platelet-derived growth factor-BB (PDGF-BB) and TGF-β1 concentrations using ELISA-kits (R&D Systems Inc., Minneapolis, MN; USA).

### Anaesthesia and medication

After premedication with either 0.5 mg/kg xylazine (Xylavet®, CP-Pharma, Burgdorf, Germany, IV) or 3.5 μg/kg dexmedetomidine (Dexdomitor®, Zoetis, Berlin, Germany) all horses were anaesthetized with 0.05 mg/kg midazolam (Midazolam-ratiopharm® 15 mg/3 ml, ratiopharm GmbH, Ulm, Germany) and 2.2 mg/kg ketamine (Narketan®, CP-Pharma, Burgdorf, Germany, IV). Anaesthesia was maintained with isoflurane (Isofluran® CP, CP-Pharma, Burgdorf, Germany) in 100% oxygen in combination with a constant rate infusion of 1 mg/kg/h xylazine or 7 μg/kg/h dexmedetomidine. intravenous fluids (lactated Ringer’s solution, Ringer-Laktat-Lösung®, B. Braun Melsungen AG, Germany) were administered at a rate of 10 ml/kg/h. Following induction of anaesthesia and tracheal intubation, the horses were positioned in dorsal recumbency and ventilated immediately. For ventilation a pressure limited and pressure cycled large animal ventilator (Vet.-Tec. Model LAVC 2000 J.D. Medical Distributing Company Phoenix, Phoenix, AZ, USA) was used. Horses were ventilated using intermittent positive pressure ventilation (IPPV) with peak inspiratory pressure of 25 cm H_2_O. Respiratory rate (f_R_) was adjusted to maintain PaCO_2_ between 40 and 45 mmHg.

### PRP preparation

Notwithstanding the application description for preparation of PRP ad modum ACP® we performed centrifugation at 1500 rpm (352 G) for 5 min but decided to waive the use of any anticoagulant in accordance to the manufacturers’ reference regarding usage of ACP® within 30 min after blood collection. All other preparation steps strictly followed the manufacturers’ application description.

Preparation of PRP ad modum E-PET strictly followed the instructions provided by the manufacturer including usage of ACD-A (acid-citrate-dextrose solution) as anticoagulant and 8 ml of a proprietary Harvest Solution for recovery of the platelets. Neither ACP® nor E-PET preparations underwent activation after processing.

### Complete blood count

Haematological analysis of fresh ACP®, E-PET and NaCl (control) aliquots were carried out using the ADVIA 120 haematology system (Siemens Healthcare GmbH, Erlangen, Germany).

### Polypeptide growth factor analysis

Double determining of the concentrations of PDGF-BB and TGF-β1 was performed by ELISA (Human PDGF-BB Quantikine® SixPak and Human TGF-β1 Quantikine® SixPak, R&D Systems Inc., Minneapolis, MN, USA) according to the manufacturers’ instructions.

### Statistical analysis

Statistical analysis was performed with SAS® software, version 9.4, for Windows (SAS Institute, Cary, NC, USA). Both for comparison with baseline values and for comparison of ACP® and E-PET, tests for paired data were applied since ACP® and E-PET-measurements were derived from the same animals. For comparison of variance, the absolute deviation from the mean was calculated, and groups were then compared using tests for paired data. Groups were compared using the two-sided Student’s t-test for normally distributed data and the two-sided Wilcoxon test for non-normal data. Data was defined as normally distributed if −1 ≤ skewness ≤ 1. Correlation was calculated with the Pearson correlation coefficient. PDGF-BB and TGF-β1 each were measured twice using an ELISA-kit as described above. The mean of the two measurements was used for analyses. To assess reliability of these measurements, intraclass correlations were computed [ICC(2, 1), ICC (2, k)].

All inferential statistics were intended to be exploratory (hypotheses generating), not confirmatory, and were interpreted accordingly. Therefore, no adjustment for multiple testing was applied. The local significance level was set to 0.05. No formal power evaluation was performed due to the explorative character of the study.

## Results

### Complete blood count of whole blood

Regardless of the conditions of sampling all values proved to be within the reference range of each parameter, but CBC data revealed lower concentrations (*p* = 0.0098) for PLT when taken under general anaesthesia (mean (M): 121 × 10^3^ / μl, 95% confidence interval (CI) ranging from 101 to 141 × 10^3^ / μl) compared to samples obtained from standing, non-sedated horses (M: 146 × 10^3^ / μl, CI: 116 to 170 × 10^3^ / μl). This was also true for WBC (*p* < 0.0001; M: 5.73 × 10^3^ / μl, CI: 4.57 to 6.88 × 10^3^ / μl) and RBC (*p* = 0.0027; M: 7.16 × 10^6^ / μl, CI: 6.68 to 7.63).

### Complete blood count of ACP and E-PET preparations

Both preparation protocols resulted in a significant increase of PLT values as compared to CBC data of whole blood samples obtained under general anaesthesia which was also true for WBC counts detected in E-PET samples but not for WBC and RBC in ACP® and RBC in E-PET.

A mean of 204 × 10^3^ thrombocytes / μl (*n* = 8, CI: 175 to 233) represented a 1.7 fold concentration of PLT in ACP® compared to whole blood concentrations under general anaesthesia (*p* = 0.0002) while for E-PET a 2.7 fold concentration of thrombocytes (*n* = 9, M: 327 × 10^3^ / μl, CI: 184 to 471) could be detected (*p* = 0.01). This difference between the preparations proved to be not significant (*p* = 1, *n* = 5). With a mean of 0.57 × 10^3^ cells / μl (*n* = 8, CI: 0.35 to 0.80) ACP® preparations reached 9.94% of the WBC count of whole blood taken under general anaesthesia. This was significantly different from WBC counts in E-PET (*p* < 0.0001) which revealed a 2.1 fold concentration (*n* = 9, M: 12.12 × 10^3^ cells / μl, CI: 10.42 to 13.82). This was also true for RBC counts with a mean of 0.04 × 10^6^ / μl (*n* = 8, CI: 0.03 to 0.06) in ACP® preparations representing 0.5% of baseline counts compared to E-PET preparations (*n* = 5, *p* > 0.0062) with a mean RBC count of 4.34 × 10^6^ / μl (*n* = 9, CI: 3.19 to 5.49) which proved to be 60% RBC counts in whole blood under general anaesthesia. The higher level of statistical dispersion in E-PET data for all three parameters (PLT, WBC and RBC) when compared to ACP® preparations was underlined by lower standard deviation values for the latter as given in Table 1.

**Table 1:** PLT, WBC and RBC concentrations (mean ± SD) in donor source and processed PRP preparations taken from equine donors

	Wb	Wba / Basline	ACP®	E-PET
PLT				
n	15	15	8	9
Mean (10^3^/μl)	143.53	121.33	204.25	327.78
SD	49.27	35.35	34.87	186.93
PWba (%)	-	-	168.34	270.15
WBC				
n	15	15	8	9
Mean (10^3^/μl)	8.87	5.73	0.57	12.12
SD	2.48	2.08	0.27	2.21
PWba (%)	-	-	9.94	211.52
RBC				
n	15	15	8	9
Mean (10^6^/μl)	8.16	7.16	0.04	4.34
SD	1.19	0.86	0.02	1.49
PWba (%)	-	-	0.56	60.61

*Wb* whole blood, *wba* whole blood under general anaesthesia, *PWba* mean percentage of cells calculated against whole blood under general anaesthesia (Baseline), *PLT* platelets, *WBC* white blood cells, *RBC* red blood cells

### Quality of growth factor analysis

Growth factor analysis via ELISA showed a high reliability for both single measurements as well as mean values calculated from double determinations. Double determined PDGF-BB values of all samples revealed an intraclass correlation of 94% with a mean difference between both measurements of 18.95 pg/ml (*n* = 152), demonstrating the accuracy of the protocol used. This was also applicable to TGF-β1 values which revealed an intraclass correlation of 89% and a mean difference between both measurements of 195.94 pg/ml (*n* = 104).

### Growth factor content in PRP preparations

Higher PDGF-BB concentrations resulted from E-PET protocols when compared to ACP®. With a mean of 3569 pg/ml (CI: 2342 to 4797) PDGF-BB concentrations of E-PET exceeded 2.8 fold the mean concentration detected in ACP® samples (M: 1276 pg/ml, CI: 1104 to 1449). The higher statistical dispersion of E-PET-generated PDGF-BB data proved to be significant (*p* = 0.03).

TGF-β1 concentrations (M: 5086 pg / ml, CI: 4554 to 5619) found in ACP® were not comparable to concentrations reached using E-PET due to technical limitations of the utilized ELISA-kit in this specific setting (Table 2). Following the manufacturers’ instructions, E-PET samples, based on equine whole blood, strongly produced red-brown colored precipitates. This phenomenon was limited to E-PET preparations and did not occur in any other setting of this study. The resulting values – with exception of six data sets from test animal 1 and three data sets from test animal 4 - did not achieve the limit of detection (1248 pg/ml). With regard to these limitations the authors decided to exclude the complete TGF-β1 data calculated for E-PET preparations.

**Table 2:** Concentrations of growth factors PDGF-BB and TGF-ß1 (mean ± SD) in PRP preparations

	*N* = 13	*N* = 9
	ACP®	E-PET
PDGF-BB (pg/ml)	1276.74 (±285.41)	3569.82 (±1597.23)
TGF-ß1 (pg/ml)	5086.98 (922.03)	n.m.

*PDGF-BB* platelet derived growth factor –BB, *TGF-ß1* transforming growth factor ß1, *n.m.* not measured

### Growth factor content over storage period

**Fig. 1:**
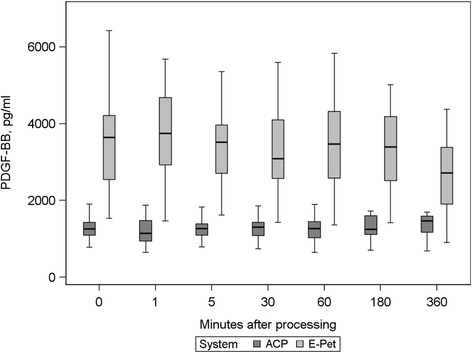
PDGF-BB concentrations over time in ACP® and E-PET preparations

**Fig. 2:**
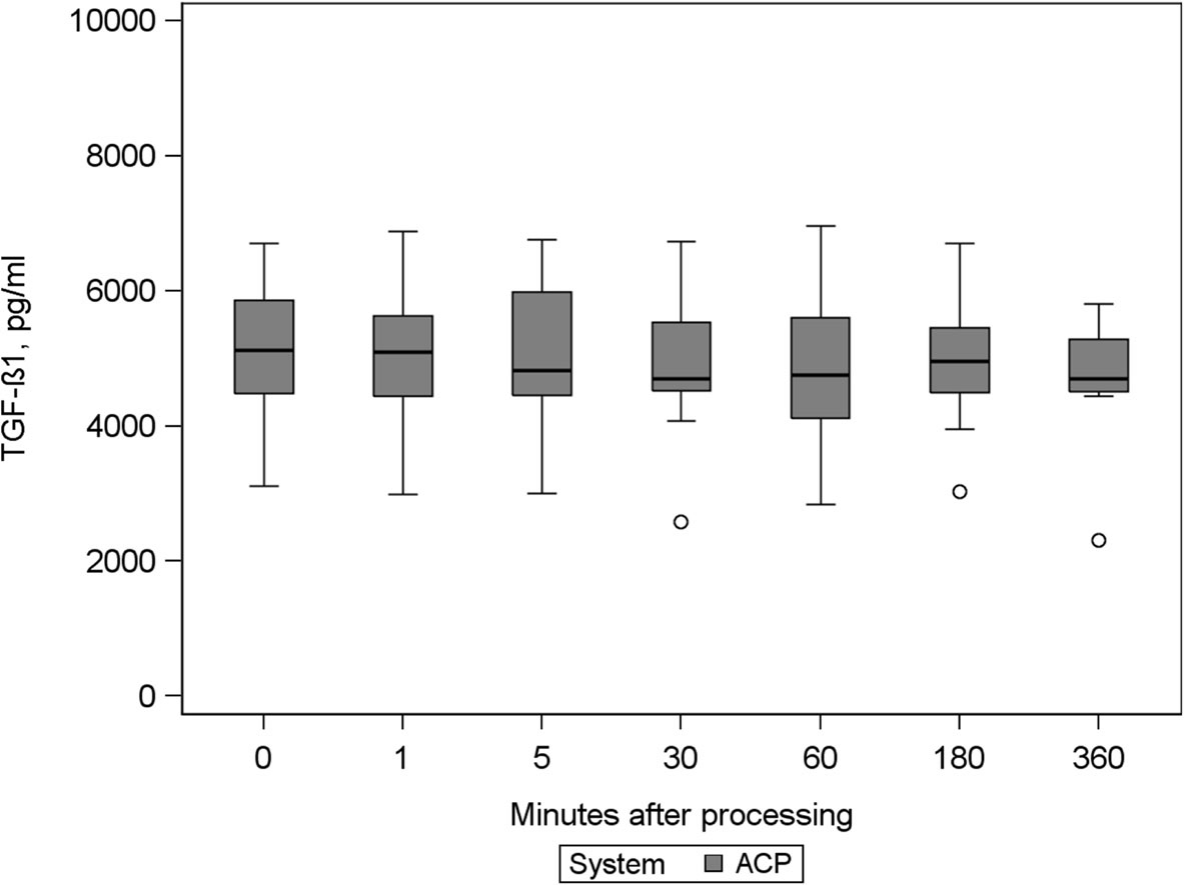
TGF-ß1concentrations at all measurement points in ACP® preparations

Both protocols revealed stable PDGF-BB concentrations for a period of 360 min after preparation (Fig. 1). This was also true for TGF-β1 concentrations in ACP® (Fig. 2) but could not be proven for E-PET preparations due to the technical limitations mentioned above.

Mean deviations from the baseline of PDGF-BB concentrations in ACP® (1276 pg/ml), calculated for the different points in time at which samples were taken, ranged from −49 to 97 pg/ml without being significant. On no account was the baseline standard deviation value (285 pg/ml) reached. Calculations for E-PET concentrations of PDGF-BB revealed mean deviations from baseline (3569 pg/ml) ranging from −296 to 76 pg/ml which proved not to be significant. In this respect, again standard deviation value of baseline measurements (1597 pg/ml) was not reached.

This was also true for TGF-β1 content of ACP® preparations showing mean deviations from baseline between −234 and 33 pg/ml calculated for the different sampling time points. This range also did not reach the standard deviation of baseline measurements (922 pg/ml).

### Control

CBC values as well as growth factor content of physiological sodium chloride solution (control group) did not reach the limit of detection.

### CBC and growth factor concentrations in relation to whole blood source

There was minor to no indication that CBC values in PRP preparations correspondend to data from whole blood taken under general anaesthesia. PLT and WBC counts in ACP® showed no significant correlations while RBC counts revealed a negative correlation (*r* = −0.84) which we found to be significant (*p* = 0.007). This was also true for haemoglobin and packed cell volume. In case of E-PET preparations no correlation of PLT counts to thrombocyte concentrations in the donor sample could be detected (*r* = −0.01, *p* = 0.96) while RBC values showed a moderate positive correlation with the donor concentrations (*r* = 0.68, *p* = 0.04) with significant RBC count correlations.

No correlations between evaluated cytokine concentrations and PLT, WBC and RBC counts of donor samples could be detected for ACP® preparations. This was also true for PDGF-BB concentrations in E-Pet.

### Growth factor concentrations in relation to CBC of PRP preparation

In ACP® preparations no significant correlation between PDGF-BB concentrations and PLT, WBC and RBC counts became obvious. No correlation could be detected for TGF-β1 values with regard to PLT and RBC counts, but a strong relationship to WBC counts was detected (*r* = 0.79, *p* = 0.01).

In contrast, PDGF-BB concentrations in E-PET strongly correlated with PLT counts of these preparations (*r* = 0.84, *p* = 0.009) which was not true for RBC values. Furthermore, a strong negative correlation with WBC values could be detected (*r* = −0.74) which proved to be significant (*p* = 0.03) (Table 3).

**Table 3:** Pearson correlation between growth factor content and CBC of PRP preparations

	PDGF-BB		TGF-ß1
	ACP®	E-PET	ACP®
PLT	*r* = 0.51 (*p* = 0.19)	*r* = 0.84 (*p* = 0.009)	*r* = − 0.26 (*p* = 0.52)
WBC	*r* = 0.24 (*p* = 0.56)	*r* = − 0.74 (*p* = 0.03)	*r* = 0.79 (*p* = 0.01)
RBC	*r* = 0.27 (*p* = 0.51)	*r* = 0.03 (*p* = 0.94)	*r* = − 0.26 (*p* = 0.52)

*r: *Pearson correlation coefficient, *p:* *p*-value (local significance level: 0.05)

## Discussion

The current study aimed to report on the short term stability of PDGF-BB and TGF-β1 in PRP processed from whole blood of equine donors. The findings revealed stable concentrations of growth factors in both, pure PRP following the ACP® protocol (a plasma based tube method) as well as – in case of PDGF-BB - in leukocyte-rich PRP resulting from E-PET protocols (a gravitiy filtration method) at room temperature over a period of at least six hours after processing. This is in line with recent findings by Anitua and colleagues [18] who stated growth factor stability in eye drops generated by the PRGF/Endoret® protocol at room temperature over a 72 h time span for a variety of different cytokines including PDGF-AB and TGF-β1 . However, our findings contradict other recommendations on PRP application which recommended applying immediately after processing [8]. It seems appropriate to address a wider range of protocols, compositions (i.e. composites with biomaterials and others) and conditions like application mode including activation and indication of PRP formulations to get a more precise impression of the individual kinetics of growth factors in a PRP setting. Thus, for example Kleinheinz and colleagues found an enhancement of VEGF_165_ (vascular endothelial growth factor) half-life from 90 min for free VEGF_165_ up to 48 h when embedded into a collagen matrix [20]. Presuming different half-life data of growth factors in dependence on the respective preparation and setting it seems to be essential to estimate cytokine stability over time not only for the main classifications of liquid platelet-rich products (P-PRP, L-PRP) but also for each individual preparation protocol currently in use. This will enable the practitioner to more precisely plan the PRP-based therapeutic approach in different indications. Particularly, but not limited to perioperative application, the ability to use PRP over an extended time span after preparation provides more flexibility during the whole process from blood sampling to final application by allowing a spatial and temporal separation of processing and application.

In this study whole blood samples for generating PRP were taken under general anaesthesia. This basic condition represents a typical setting in veterinary medicine due to a lack of patients’ compliance during the subsequent application procedure. Especially in case of intraarticular application of PRP in small animal medicine this condition will help for example to avoid iatrogenic cartilage lesions caused by patient movement under application. It may also be considered for application in human as well as veterinary medicine if used in a surgical setting. PLT, WBC and RBC concentrations of these samples were significantly lower than CBC counts in standing, non-sedated horses but still proved to be within the range of normal (physiological) values as expected [21, 22]. Individual variations in CBC counts between horses were larger using the E-PET protocol and might have been influenced by age and gender [23]. Daily as well as temperature-associated variations of PRP preparations have also been described [24]. These factors were not considered in the current study design. However, the authors assume that the back-flushing step during preparation with the filter-based E-PET system is by contributed to individual variations.

As part of a broader study we chose two different types of PRP preparation protocols intended to generate a PRP containing low to none WBC content – pure PRP (P-PRP) - in case of ACP® and a leukocyte-rich PRP (L-PRP) following the E-PET protocol. This differentiation represents the current consensus on the classification of PRP preparations used in liquid form [25]. The former preparation system is well established in human and veterinary companion animal medicine while the latter was originally developed for the use in equids. Deviating from the manufacturers’ recommendations for equine applications, ACP® was processed using higher relative centrifugation forces (rcf) during the single spin process for a maximum reduction of WBC counts. According to Kissich and colleagues [26] the ACP® preparation protocol recommended by the manufacturer for equine applications (rpm: 1100, rcf: 189 g, 5 min) revealed 1.5 fold increase in PLT counts to baseline coming along with a reduction of WBC concentrations to 11.9% of baseline counts. A higher rcf protocol – which is also in accordance to current human application guidelines - (rpm: 1500, rcf: 352 g, 5 min) resulted in a 1.2 fold increase in PLT concentrations along with a reduction of WBC counts to 1.3% of baseline. Kissich and coworkers in their study took whole blood samples as source from standing, non-sedated horses. To come close to the definition of pure PRP we decided to use this latter spin-protocol. In accordance to the preparation protocols used by most of the practitioners - as experienced by the authors – no anticoagulant was used. This protocol resulted in a greater increase of PLT counts (1.7 fold) but only a slight decrease in WBC concentrations compared to the recommended procedure. With regard to this ACP® procedure, the impact of the anticoagulant on these findings should be negligible as waiving the anticoagulant is to be expected to rather impair physiological and structural integrity of platelets leading to reduced PLT counts [27]. However, this was not confirmed by this study. Inconsistencies in resulting CBC data is underlined by a study from Hessel and colleagues with CBC counts being different despite using same protocol as the Kissich group [28].

Along with a 2.1 fold increase in WBC counts from baseline the gravity filtration-based E-PET system revealed a 2.7 fold increase of PLT content when compared to the whole blood source when strictly following the manufacturers’ recommendations including use of ACD-A (anticoagulant citrate dextrose solution A) as anticoagulant. The lack of significance of the difference in enrichment when compared to ACP® data is probably due to the small number of comparable samples (*n* = 5). However, mean PLT counts (327.78 × 10^3^/μl) markedly deviated from mean PLT concentrations (533 × 10^3^/μl) reached by Hessel and colleagues [28] as well as by Textor and colleagues (650 × 10^3^/μl) [29]. These two groups used the E-PET protocol but performed whole blood sampling from non-anaesthetized donors. Interestingly, despite these inconsistencies regarding the CBC data in the processed PRP formulations, mean PDGF-BB concentrations resulting from E-PET processing in our study (3569.82 pg/ml) approximately matched values reached in activated E-PET preparations by Textor and colleagues (mean: 3811 pg/ml). This underlines current discussions on the definition of optimal dose or concentration of platelets and growth factors in PRP which is agreed to be another important topic in PRP research at present [30, 31]. Variables influencing this phenomenon remain questionable. Considering a single freeze-thaw cycle prior to growth factor analysis as part of the design of the current study and the Hessel group, respectively, both preparations must be considered thermally activated at the time of ELISA processing. Early studies have shown that, with regard to PDGF, the freezing procedure itself does not impair the resulting measurable cytokine concentrations [32]. Thus, sampling conditions of whole blood apparently remain the only difference in E-PET processing.

However, statistical correlations calculated revealed no impact of PLT counts in whole blood on the resulting thrombocyte content and PDGF-BB concentrations of L-PRP preparations ad modum E-PET. Furthermore, due to a lack of significance of correlations between RBC and WBC counts in the whole blood sample and the platelet-rich product as well as between these cell concentrations and resulting PDGF-BB values, a convincing impact of CBC counts of whole blood samples on resulting CBC counts and PDGF-BB content in this L-PRP preparation could not be shown. Consequently, we consider the differentiation of the donor condition in “standing, non-sedated” and “under general anaesthesia” not to be relevant with regard to resulting CBC counts and PDGF-BB content of this L-PRP protocol. If other anaesthetic protocols and resulting splenic RBC sequestration for example may have a significant impact on cell content and function of whole blood source [21, 22,﻿ 33] as well as on growth factor content of L-PRP preparations has to be subject to further studies.

In contrast to this, as expected, we found a strong correlation of resulting cytokine values with the E-PET preparations’ PLT counts. However, these findings were accompanied by a significant negative correlation of WBC content of the processed platelet-rich product with resulting PDGF-BB concentrations. Due to the leukocytes’ ability to produce new growth factors after an initial release [34], a positive correlation between PDGF-BB content and WBC counts should readily be anticipated, but could not be confirmed by our results. The identification of variables causing this negative dependency is beyond the design of this study.

In case of ACP® preparations, no impact of CBC counts of the donor sample on cytokine concentrations after processing was found. The strong negative correlation between RBC values of sample and preparation underlines the ability of this P-PRP protocol to nearly eliminate RBCs from the final preparation. Unlike the E-PET protocol, no reliable dependencies of resulting PDGF-BB concentrations from the preparations’ CBC could be defined. This was also true for TGF-β1 with the exception of WBC counts significantly influencing the resulting content as expected when considering leukocytes as a vital source of this cytokine [35].

Based on the results of this study defining an unambiguous pattern of dependencies between cytokine content of PRP preparations and CBC concentrations of the donor source is not possible. Even the strong correlation between PLT values of E-PET preparations and resulting PDGF-BB content is contradicted by the results of other investigators [28, 29] who plotted approximately similar PDGF-BB concentrations coming from nearly two times higher PLT counts while applying this same protocol.

## Conclusion

Both preparation protocols representing L-PRP and P-PRP revealed stable growth factor concentrations over at least a six hour time span at room temperature. With regard to the activation step by a freeze-thaw cycle prior to measurement– resulting in the release of all growth factors - this may be addressed as a stable growth factor potential over the time span considered. The correlations calculated in this study may indicate the absence of any impact of the respective preparations’ PLT counts on resulting cytokine values in case of P-PRP preparations while for L-PRP a strong correlation occurred. Due to inconsistent patterns, these findings have to be addressed as tendencies. Additional studies implicating a further standardization of processing protocols are necessary to identify consistent impact on cytokine content after PRP processing.

## Additional file

Additional file 1: Computer spreadsheet containing data from all studies. (XLSX 23 kb)

## Abbreviations

ACD-A: Acid-citrate-dextrose solution
ACP®: Autologous conditioned plasma
CBC: Complete blood count
CI: Confidence interval
E-PET: Equine platelet enhancement therapy
IPPV: Intermittend positive pressure ventilation
L-PRP: Leukocyte-rich platelet-rich plasma
M: Mean (statistical)
NaCl: Sodium chloride solution
NRC: National research council (US)
PDGF-BB: Platelet-derived growth factor-BB
PLT: Platelets (thrombocytes)
P-PRP: Pure platelet-rich plasma
PRP: Platelet-rich plasma
RBC: Red blood cells
rcf: Relative centrifugation force
rpm: Rounds per minute
TGF-ß1: Transforming growth factor-ß1
VEGF: Vascular endothelial growth factor
WBC: White blood cells
